# Acute Respiratory Distress Syndrome due to *Mycoplasma pneumoniae* Misinterpreted as *SARS-CoV-2* Infection

**DOI:** 10.1155/2021/5546723

**Published:** 2021-05-29

**Authors:** Carlos Metz, Torben Rixecker, Sebastian Mang, André Becker, Alexander Maßmann, Sören L. Becker, Cihan Papan, Barbara Gärtner, Frederik Seiler, Guy Danziger, Robert Bals, Philipp M. Lepper

**Affiliations:** ^1^Department of Pneumology, Allergology and Critical Care Medicine, Saarland University, Homburg/Saar, Germany; ^2^Department for Diagnostic and Interventional Radiology, Saarland University Medical Center, Homburg/Saar, Germany; ^3^Center for Infectious Diseases, Institute of Medical Microbiology and Hygiene, Saarland University, Homburg/Saar, Germany

## Abstract

**Background:**

In 2020, a novel coronavirus caused a global pandemic with a clinical picture termed COVID-19, accounting for numerous cases of ARDS. However, there are still other infectious causes of ARDS that should be considered, especially as the majority of these pathogens are specifically treatable. *Case Presentation*. We present the case of a 36-year-old gentleman who was admitted to the hospital with flu-like symptoms, after completing a half-marathon one week before admission. As infection with SARS-CoV-2 was suspected based on radiologic imaging, the hypoxemic patient was immediately transferred to the ICU, where he developed ARDS. Empiric antimicrobial chemotherapy was initiated, the patient deteriorated further, therapy was changed, and the patient was transferred to a tertiary care ARDS center. As cold agglutinins were present, the hypothesis of an infection with SARS-CoV-2 was then questioned. Bronchoscopic sampling revealed *Mycoplasma (M.) pneumoniae*. When antimicrobial chemotherapy was adjusted, the patient recovered quickly.

**Conclusion:**

Usually, *M. pneumoniae* causes mild disease. When antimicrobial chemotherapy was adjusted, the patient recovered quickly. The case underlines the importance to adhere to established treatment guidelines, scrutinize treatment modalities, and not to forget other potential causes of severe pneumonia or ARDS.

## 1. Introduction

A novel coronavirus, SARS-CoV-2, caused a global pandemic with a clinical picture termed COVID-19 and accounted for numerous cases of ARDS in early 2020 [1]. To date, there is no convincing evidence for a specific medical therapy for SARS-CoV-2. Most European hospitals prepared for a surge of these patients. COVID-19 leads to a systemic disease primarily affecting the lung. Approximately 15–42% of COVID-19 patients develop ARDS (CARDS) [1–3]. However, there are still other infectious causes of ARDS that should be considered, especially, as the majority of these pathogens are specifically treatable. The case presented here describes a severe infection with *M. pneumoniae* leading to ARDS in an adult, complicated by a delay in diagnosis and effective therapy as COVID-19 was suspected.

## 2. Case Presentation

On the 14th of March 2020, a 36-year-old gentleman presented at the emergency unit of a nearby hospital with fever, dry cough, and head and limb aches that started three days earlier. In good physical condition and otherwise healthy, he had completed a half-marathon the week before, but on admission to another hospital, he presented with reduced general condition and shortness of breath. Physical examination revealed crackles in the right upper lobe upon auscultation. Medical, family, and psychosocial history was completely unremarkable.

As SARS-CoV-2 was suspected, he was immediately admitted to the ICU. He received microbiological and virological sampling as well as chest X-ray and thoracic CT. A calculated antimicrobial chemotherapy with piperacillin/tazobactam (3 × 4.5 g/d i.v.) and clarithromycin (2 × 500 mg/d i.v.) was started. Polymerase chain reaction (PCR) for SARS-CoV-2 was negative, as were the results for influenza, *Mycobacterium tuberculosis*, and *Legionella* spp. After 2 days, the antimicrobial regimen was changed to meropenem (3 × 1.0 g/d i.v.), linezolid (2 × 600 mg/d i.v.), and fosfomycin (3 g/d i.v.) due to persistently elevated inflammatory parameters and further clinical deterioration. The assumption of the patient having COVID-19 was maintained. Imaging of the lungs revealed a diffuse interstitial reticular pattern, multilobular patchy ground-glass opacification, and consolidation of the right upper lobe.

After a week on high-flow oxygen, he deteriorated and was intubated at an oxygenation index (P_a_O_2_/F_i_O_2_ ratio) of 130. Despite proning, he deteriorated further (hypercapnia with respiratory acidosis and hypoxemia with a P_a_O_2_/F_i_O_2_ ratio 108), needing higher doses of vasopressors, and our hospital's extracorporeal membrane oxygenation (ECMO) team was called on the 22^nd^ of March 2020 to transfer the patient. The patient was reported as having COVID-19 and moderate ARDS with respiratory acidosis.

The ECMO team set out to transfer the patient under COVID-19 personal protective equipment (PPE). In the external hospital, after reviewing the laboratory and imaging findings, as well as the ventilator settings, the team leader decided against implanting an ECMO on site, and the patient was transferred under COVID-19 precautionary measures. Bronchoscopy with bronchoalveolar lavage (BAL) was performed for microbiological sampling and to further elucidate the hypothesis of an infection with SARS-CoV-2. Additionally, a naso- and oropharyngeal swab for SARS-CoV-2 was obtained.

**Figure 1:**
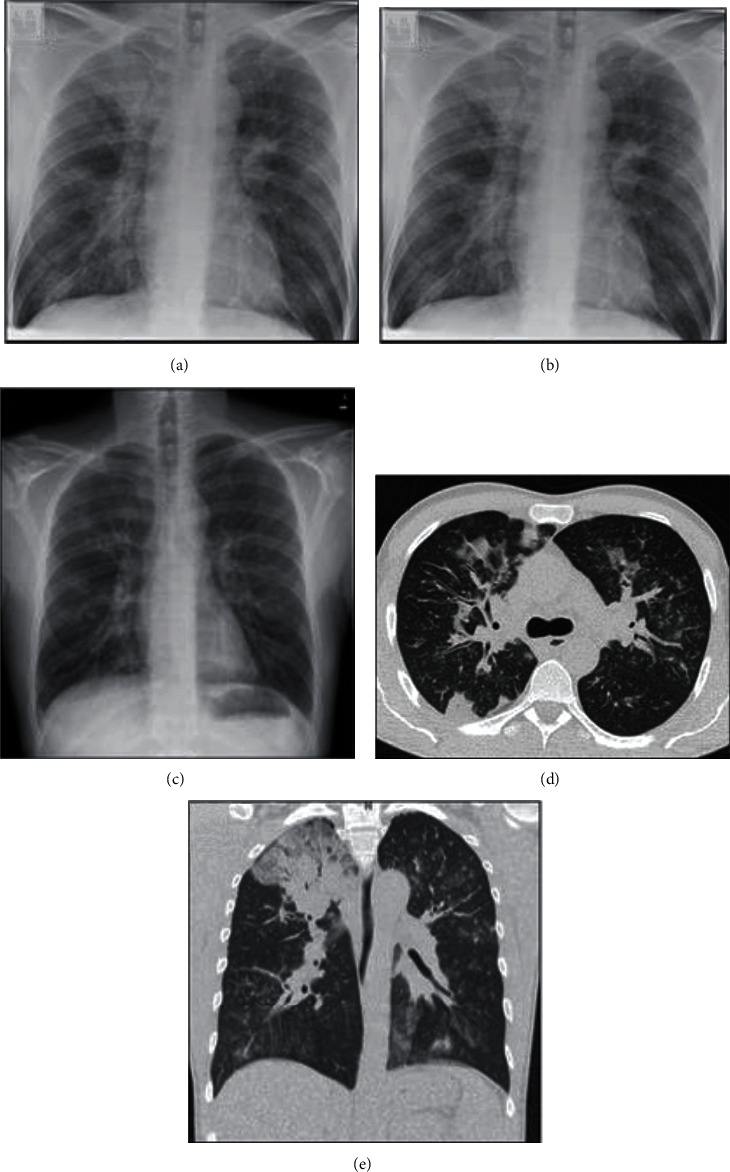
(a) Chest radiograph on the day of admission (March 14, 2020) to an external hospital shows consolidation predominantly in the right upper lobe, vague ill-defined opacities in the right lower lobe and left hilar region, and a diffuse interstitial pattern combined with bronchial wall thickening. (b) Chest X-ray on the day of admission to a tertiary care hospital depicts progressive pneumonia characterized by diffuse reticular and nodular patterns (March 22). (c) Chest X-ray shortly after discharge from the ICU (April 2) shows almost complete regression of previous infiltrations. The patient did not need supplementary oxygen at that time. (d, e) Computed tomography of the chest on March 16^th^ confirms consolidation of the right upper lobe and reveals multifocal, patchy consolidations, ill-defined airspace infiltrates, and ground-glass opacifications. Additional centrilobular nodular appearance and thickening of the bronchovascular structures are present.

Chest radiographs were reviewed, and a new chest X-ray was made (Figure 1). Imaging showed mainly right-sided pneumonia, not typical for COVID-19, and the hypothesis of COVID-19 was abandoned. Antimicrobial therapy was changed, as atypical pneumonia was suspected. Meropenem (then given continuously i.v., monitored by determination of serum levels) was continued for a total of 7 days, and clarithromycin (2 × 500 mg/d) was added to the antimicrobial regime again (Table 1). Remarkably, for a young and otherwise healthy individual, the patient had elevated bilirubin and LDH levels, with diminished haptoglobin and macrocytic hyperregenerative anemia (hemoglobin 5.3 g/dL at a s_cv_O_2_ of 67%) on admission, demonstrating hemolysis. Coombs' test revealed cold agglutinins the same day.

Microbiological results from a BAL (March 22^nd^) were negative for *SARS-CoV-2*, *Pneumocystis jirovecii*, *Bordetella pertussis* and *B. parapertussis*, *Chlamydophila pneumoniae*, *Haemophilus influenzae*, *Legionella pneumophila*, *Moraxella catarrhalis*, and *Streptococcus pneumoniae*; however, the patient tested positive for *Mycoplasma (M.) pneumoniae*. Thus, a diagnosis of mycoplasma-related ARDS with cold agglutinin disease was made. The patient was in total substituted with 4 units of packed red blood cells and received only warm infusions. Ventilator support was deescalated soon after change of the antimicrobial regimen, and the patient was extubated on March 25^th^ receiving noninvasive ventilator support for 4 more days with supplementary oxygen up to 40%. Meropenem was continued for a total of 7 days, and clarithromycin (2 × 500 mg/d) was added to the antimicrobial regime again (Table 1). Hemolysis improved quickly under the antibiotic regime. The patient was discharged from the ICU on March 30^th^ and was discharged home without supplementary oxygen on April 6^th^. He had no health-related complaints in a telephone interview conducted on June 18^th^.

## 3. Discussion

Under the impression of a seemingly predominant microorganism at the time of admission, a clinical diagnosis was established that was questionable according to radiologic evidence. Additionally, inappropriate management of the pulmonary infection might have favored clinical deterioration. Initial antimicrobial therapy was changed early empirically to a regimen that was much less effective for the causative microorganism, despite a microbiological workup. The initial therapy was performed according to current guidelines [4]. The change was meant to extend the spectrum; instead, it missed the causative organism. Furthermore, at the time antimicrobial therapy was changed, treatment failure was not proven.

*M. pneumoniae* is a common cause of community-acquired pneumonia, particularly in children and young adults [5]. *M. pneumoniae* is a very small bacterium without a peptidoglycan cell wall. It is a common cause of tracheobronchitis and atypical pneumonia mainly because of its adherence to respiratory cells. Infection of host cells occurs through special adhesins and an elongated polar attachment organelle [6]. Usually, the pneumonia caused by *M. pneumoniae* is mild and characterized by a dry cough or self-limiting pneumonia [7]. The rates of ICU admission ranged between 10% and 16%. With an intense epidemiological background of COVID-19 as a cause of respiratory disease leading to a surge of ICU admissions in many countries, the proper diagnosis of treatable causes for ARDS is highly important. Therefore, microbiological testing from respiratory material (bronchial aspirate) using adequate test methods, such as loop-mediated isothermal amplification (LAMP) [8] or quenching probe (QProbe) [9] methods for *M. pneumoniae* diagnosis, is highly important, especially as macrolide resistance rates are increasing in *M. pneumoniae* [10].

Severe ARDS and fatal outcomes due to *M. pneumoniae* are rare and may be the result of unclear clinical features, delayed diagnosis, inappropriate respiratory support, and/or insufficient initial treatment. If additional diagnostic measures are not confirming the suspected pathogen, alternative explanations need to be evaluated. This is especially important if a treatable cause is present, as in the case presented here. However, concomitant cold agglutinin disease is frequently described in the context of *M. pneumoniae* and usually develops upon generation of polyclonal IgM antibodies directed against I antigens on RBCs. Hemolysis can be severe but is usually self-limiting, while corticosteroids are reported to be barely effective [11].

If severe pneumonia caused by *M. pneumoniae* should be treated with corticosteroids in general remains unclear. While positive effects have been shown in children, there is a lack of prospective studies defining the appropriate dose and duration of steroid administration in fulminant ARDS with *M. pneumoniae* in adults [12].

Antibiotic therapy of *M. pneumoniae* requires agents such as macrolides or fluoroquinolones that do not target the bacterial cell wall and have good intracellular penetration. In our patient, macrolide therapy was started according to guidelines for severe pneumonia but was stopped after 2 days, and the regimen was unintentionally changed to a less effective one. These decisions might have been driven by the assumption that the patient might have an infection with SARS-CoV-2.

The gold standard for the detection of COVID-19 in symptomatic individuals is the detection of viral RNA in naso- or oropharyngeal swabs by reverse-transcriptase polymerase chain reaction (rtPCR) that can be false negative [13].

Radiologic findings alone are often not reliable for differentiating pneumonia. Moreover, coinfection with other bronchopulmonary pathogens is not uncommon. In general, radiographic findings should be used along with clinical and laboratory data to narrow the differential diagnosis. Currently, there is a threat of misinterpreting clinical pictures and lung imaging as SARS-CoV-2-induced disease. It has been suggested only recently that low-dose CT might be of equal sensitivity and specificity as rtPCR testing of nasopharyngeal swabs [14]. However, multiple infectious diseases might produce similar pictures in pulmonary imaging modalities.

At the time, the patient in the present case acquired the infection leading to ARDS, and the prevalence of COVID-19 in Germany, especially in the Federal State of Saarland, was low (2,078 confirmed cases in Germany on March [12]). In contrast, awareness of COVID-19 was high-flying.

If the prevalence of a specific infectious agent is predominant, it is very likely that the radiologic picture in fact happens to be the at the time frequently encountered infectious agent. However, the effective performance of CT for COVID-19 detection critically depends on the pretest probability for the occurrence of a disease, which in turn influences positive and negative predictive values (PPV and NPV). If the prevalence of a disease is truly low, the PPV for the disease will be low. If caregivers overestimate PPV, they might come to the wrong conclusion if no gold standard for the diagnosis of a disease exists or is accepted.

In the present case, treating physicians unintentionally created an unfavorable situation for the patient urged by erroneous assumptions. Unfortunately, the well-intentioned putative escalation of antimicrobial therapy was less effective for the causative organism.

## 4. Conclusion

The case presented here underlines the importance of adhering to established treatment guidelines, scrutinizing treatment modalities, and not forgetting other potential causes of severe pneumonia or ARDS to ensure that critically ill patients are safeguarded from common infections even in times ruled by a predominant pathogen.

## Figures and Tables

**Table 1:** Patient characteristics and laboratory parameters at various time points during infection with *M. pneumoniae*.

*Parameter*				
Gender	Male			
Age (years)	36			
Height (cm)	190			
Weight (kg)	90			
Body mass index (kg/m^2^)	24.9			
*Antimicrobial therapy (therapy started on the 14^th^ of March, the patient transferred on the 22^nd^ of March)*				
Piperacillin/tazobactam	14.03-18.03			
Meropenem	18.03-24.03			
Linezolid	17.03-22.03			
Fosfomycin	17.03-22.03			
Clarithromycin	14.03-16.03 and 22.03–03.04			
*Laboratory findings*	*Date*			
	*22^nd^ of March*	*2^nd^ of April*	*6^th^ of April*	*Normal values*
LDH (IU/L)	993	532	512	0–262
CRP (mg/L)	216	13	4.8	0.0-5.0
Bilirubin (mg/dL)	1.4	0.5	0.4	<1.2
Haptoglobin	<5	n.a.	n.a.	>5
Creatinine (mg/dL)	1.03	0.68	0.76	0.70–1.20
Sodium (mmol/L)	148	140	141	135–145
Potassium (mmol/L)	5.4	4.2	5.6	3.5–5.1
Hemoglobin (g/dL)	5.3	7.7	9.0	14.0–18.0
WBC (G/L)	16.8	6.6	7.6	3.9–10.2
Thrombocytes (T/*μ*L)	351	559	568	140–400
Fibrinogen (g/L)	354	369	420	180–400
Interleukin 6 (pg/mL)	51.5	n.a.	n.a.	<7
D-dimers (mg/L)	12.6	n.a.	n.a.	<0.50

n.a.: not available.

## Data Availability

Data can be provided on request addressed to the corresponding author. All data sharing statements are subject to conformity with German data protection legislation and rules (Datenschutzgrundverordnung-DGSVO).

## Abbreviations

ARDS: Acute respiratory distress syndrome
CAD: Cold agglutinin disease
ECMO: Extracorporeal membrane oxygenation
NPV: Negative predictive value
PPE: Personal protective equipment
PPV: Positive predictive value.
